# Brain State Decoding Based on fMRI Using Semisupervised Sparse Representation Classifications

**DOI:** 10.1155/2018/3956536

**Published:** 2018-04-19

**Authors:** Jing Zhang, Chuncheng Zhang, Li Yao, Xiaojie Zhao, Zhiying Long

**Affiliations:** ^1^State Key Laboratory of Cognitive Neuroscience and Learning & IDG/McGovern Institute for Brain Research, Beijing Normal University, Beijing 100875, China; ^2^School of Information Science & Technology, Beijing Normal University, Beijing 100875, China

## Abstract

Multivariate classification techniques have been widely applied to decode brain states using functional magnetic resonance imaging (fMRI). Due to variabilities in fMRI data and the limitation of the collection of human fMRI data, it is not easy to train an efficient and robust supervised-learning classifier for fMRI data. Among various classification techniques, sparse representation classifier (SRC) exhibits a state-of-the-art classification performance in image classification. However, SRC has rarely been applied to fMRI-based decoding. This study aimed to improve SRC using unlabeled testing samples to allow it to be effectively applied to fMRI-based decoding. We proposed a semisupervised-learning SRC with an average coefficient (semiSRC-AVE) method that performed the classification using the average coefficient of each class instead of the reconstruction error and selectively updated the training dataset using new testing data with high confidence to improve the performance of SRC. Simulated and real fMRI experiments were performed to investigate the feasibility and robustness of semiSRC-AVE. The results of the simulated and real fMRI experiments showed that semiSRC-AVE significantly outperformed supervised learning SRC with an average coefficient (SRC-AVE) method and showed better performance than the other three semisupervised learning methods.

## 1. Introduction

Functional magnetic resonance imaging (fMRI), which measures brain activity by detecting changes in blood oxygenation level-dependent signals, is a powerful technique for indirectly investigating the neural activity in the brain. Recently, multivariate classification techniques have been widely applied to fMRI data to decode brain states from observed brain activities [1]. Compared with the traditional univariate analysis methods, multivariate supervised-learning techniques are able to reveal the neural mechanism that is discriminative to different brain states [2].

Among the various multivariate supervised-learning classification techniques, sparse representation-based classification (SRC) exhibits a state-of-the-art classification performance and is robust against noise. SRC has attracted increasing attention and achieved promising results in many areas, for example, image [3], digit, and texture classifications [4, 5]. SRC represents the test sample using an overcomplete dictionary whose base elements are the training samples. If sufficient training samples are available from each class, SRC will be possible to represent the test samples as a linear combination of the training samples from the same class. Although various supervised-learning classification techniques that included support vector machine (SVM), logistic regression, naïve Bayesian, and deep neural networks were applied to brain state decoding of fMRI data [6–9], SRC has seldom been applied to fMRI-based brain state decoding due to the various variabilities in fMRI data, such as complex and high noises and the delay of hemodynamic response. Given the promising outcomes of SRC in other research fields, it is necessary to explore the effective use of SRC in fMRI analysis.

SRC is a type of supervised-learning method that must be trained using labeled samples. If the labeled training data are insufficient, the performance of the trained classifier cannot be guaranteed. Because the collection of human fMRI data is restricted by the high cost of experiments and is highly constrained by the limited amount of time during which a participant can safely remain in the scanner, it is challenging to collect a large amount of labeled training data for a participant. To solve the insufficiency of labeled training data, semisupervised learning was developed to train the classifier using both labeled training data and unlabeled data. Many machine learning studies have found that unlabeled data, in conjunction with a small amount of labeled data, can produce a considerable improvement in the learning accuracy [10, 11].

Various semisupervised-learning algorithms have already been developed over the past decade, including self-training [10, 12], cotraining [13], transductive support vector machine [14], graph-based algorithms [11], and generative models [15]. Among these methods, self-training is a simple and effective model and is less time-consuming than the other models [16]. Self-training gradually updates the labeled training data by using test samples with the most confident predictions step by step to improve the performance of the traditional supervised learning algorithm. In contrast to most conventional classifications that are usually divided into two independent steps, that is, training and testing, SRC does not have a training process, and all test data are adaptively represented by all the training samples in the dictionary. Therefore, SRC has an adaptive characteristic [17] and does not need to be retrained as the training data are gradually enlarged. Therefore, self-training can be easily combined with SRC. Thus far, one study proposed a type of semisupervised SRC method for EEG in brain-computer interface application by combining self-training learning and SRC [17]. This method simply updates all tested data without estimating the confidence of the predictions, which may result in performance degeneration due to many false predictions.

In addition, a few semisupervised machine learning methods have recently been proposed for fMRI data analysis. Plumpton et al. proposed a naïve random subspace ensemble strategy using linear classifiers [18]. This method is time-consuming and can easily be affected by testing samples with inaccurate predictions. Plumpton (2014) further proposed random subspace ensemble of online linear discriminant classifier (RSE-OLDC) that updates only the predicted labels with a high confidence rather than all predicted labels [19]. However, two parameters, that is, the subspace scale and the number of individual classifiers in RSE-OLDC, may vary with different datasets, and the random selection of feature subsets may induce some fluctuations in the classification results. Due to the low signal-to-noise ratio (SNR) and low sample-to-feature ratio of fMRI data, the introduction of a few incorrect sample labels may heavily affect the classification performance. Therefore, a robust and effective semisupervised learning method is essential for brain state decoding based on fMRI data.

This study aimed to investigate how to improve SRC and effectively applied SRC to fMRI-based decoding. Zou et al. (2015) proposed a local sparse representation-based nearest neighbor (LSRNN) classifier that averaged the *k* largest sparse coefficients in each class and assigned the label of the class with the maximum average sparse coefficient to the testing sample [20]. It was demonstrated that class-specific sparse coefficients could be utilized to improve the performance of classification. Based on the previous study, this study proposed the semisupervised SRC with an average coefficient (semiSRC-AVE) method that performed the classification using the average coefficient of each class instead of the reconstruction error and selectively updated the training dataset using new testing data with high confidence to improve the performance of SRC. The results of the simulated and real fMRI data both demonstrated that semiSRC-AVE exhibited a more stable and better performance than the supervised SRC with an average coefficient (SRC-AVE) method. Compared to the other three semisupervised methods, including naïve semiSRC-AVE, RSE-OLDC [19], and random subspace ensemble of online SRC-AVE (RSE-OSRC-AVE), semiSRC-AVE showed better performance in the multiclass classification and comparable performance in the two-class classification.

## 2. Related Works

For self-training, the confidence of prediction is calculated after a test sample is classified. If the confidence is higher than a threshold, the test sample and its predicted label are added to the training set and the classifier is retrained for the next test sample. The confidence of predication is critical to the self-training algorithm. An appropriate confidence measure can prevent test samples with wrong predicted label from entering into the training set. Different self-learning classifiers may use different confidence measures.

For the well-known decision tree algorithm C4.5 [21], the confidence of a prediction can be obtained from the accuracy of the leaf, that is, the percentage of correctly classified training samples from all training samples [16]. For the self-training naïve Bayesian classifier (NB), the confidence is determined by the probability of predicted class for a given test sample [16]. The self-training SVM algorithm can determine the confidence of a prediction using Plat scaling [22] that returns posterior probability of predicted class for a test sample [23].

Recently, a few self-training update strategies have been applied to fMRI-based classification. Naïve strategy does not judge the predicted labels' reliabilities and updates the classifier using the predicted naïve labels directly. Plumpton et al. (2012) applied the naïve strategy to a random subspace ensemble classifier that used the vote result of the ensemble linear discriminant classifiers as the true label and updated the classifier by adding the test data to the training set [18].

Plumpton (2014) further improved their ensemble method and proposed the new random subspace ensemble of online linear discriminant classifier (RSE-OLDC) by updating the training data using the predicted labels with a high confidence [19]. Because RSE-OLDC was used in this study, we presented a detail review on RSE-OLDC.

RSE-OLDC has two parameters that are the number of individual classifiers (*L*) and the subspace scale (*M*). Suppose that each sample has *n* features. For each training sample, *L* feature subsets are drawn independently. Each subset contains *M* < *n* features that are randomly selected from the total feature set without replacement. Therefore, *L* training datasets are generated and *L* diverse linear discriminant classifiers (LDC) [18] are generated by training each ensemble member on a different training dataset. Suppose that the training data for class *i* come from a multivariate normal distribution with the class-specific mean *μ*^*i*^ and the common covariance matrix Σ. The optimal discriminant function *g*_*i*_(*x*) of LDC for a test sample *x* is calculated by

(1) $$\begin{array}{c} g_{i}\left(\;x\;\right)=\ln P^{i}-\frac{1}{2}{\mu^{i}}^{T}\Sigma^{-1}\mu^{i}+{\mu^{i}}^{T}\Sigma^{-1}x. \end{array}$$

The test sample *x* is assigned to the class with the largest *g*_*i*_(*x*).

For each test sample, *L* feature subsets are generated in the same way as the training datasets. Each feature subset of a testing sample has the same features as the training datasets. The *L* classifiers are applied to the corresponding *L* feature subsets of a testing sample separately. The final prediction of a test sample is determined by majority vote of the *L* ensemble classifiers. Suppose that *y* is the final prediction and *y*_*i*_ is the predicted label of the *i*th classifier. Confidence of the prediction is calculated by

(2) $$\begin{array}{c} \text{confidence}=\frac{\sum_{i=1}^{L}\left\{y_{i}=y\right\}}{L}. \end{array}$$

For the next test sample, the classifiers were updated by adding the test sample with the confidence higher than a threshold to the training dataset. Plumpton chose 75% as the threshold in their study [19].

## 3. Proposed Methods

In this section, the theoretical frameworks underlying the SRC and semiSRC-AVE methods are described.

### 3.1. Sparse Representation-Based Classification

SRC aims to seek a suitable sparse solution to represent test data *y* from the whole training set [3]. Suppose that the matrix *A* = [*A*_1_, *A*_2_,…, *A*_*k*_] ∈ *R*^*N∗M*^ concatenates the *M* training samples of all *k* classes, *N* represents the feature dimension of the sample, and *A*_*i*_ represents the subset of training samples of class *i*. Let *y* be a test sample. If the training samples in the dictionary are sufficient, test sample *y* can be represented by solving the following problem:

(3) min x0s.t. y=Ax,

where *x* is a coefficient vector. The above *l*_0_-norm minimization problem is nonconvex and NP-hard. If the solution *x* is sufficiently sparse, the *l*_0_ minimization problem in (3) is equivalent to the *l*_1_ minimization problem in [24]

(4) x^=arg min x1s.t. y=Ax.

The *l*_1_ minimization problem has been broadly investigated, and various algorithms can be used to solve it [25]. In this study, the gradient projection for sparse reconstruction (GPSR) is applied due to its relatively rapid computation speed.

After the sparse coefficient vector $\hat{x}$ is estimated, the classification can be performed by

(5) $$\begin{array}{c} I\left(\;y\;\right)=\arg\underset{i}{\min}\;r_{i}\left(\;y\;\right)=\arg\underset{i}{\min}{\left\|\;y-A\delta_{i}\left(\;\hat{x}\;\right)\;\right\|}_{2}. \end{array}$$

Here, *r*_*i*_(*y*) is the representation residual error corresponding to class *i*. $\delta_{i}(\hat{x})$ is a vector whose nonzero elements are those that are associated with class *i*.

In the context of fMRI-based brain state decoding, *y* ∈ *R*^*N*^ is the fMRI volume at a time point of the testing data and *N* is the number of the spatial voxels. Each column in the matrix *A* represents the fMRI volume of the training data from one of the tasks (classes). The goal of classifier model is to determine which class the test data *y* belongs to.

### 3.2. Semisupervised SRC with an Average Coefficient (semiSRC-AVE)

For fMRI data, the hemodynamic responses have a delay of approximately 6 seconds to reach the maximum value after a short-duration stimulus. In contrast to the static face image, the fMRI volumes that respond to the same task vary greatly across different time points. Those variations may largely affect the performance of SRC in fMRI data analyses.

If a test sample belongs to a specific class, it is generally positively correlated with the training samples in the same class and should be better represented by the training samples from the class with larger positive coefficients and smaller negative coefficients compared to those from the other classes. Therefore, the average of all coefficients associated with a specific class may be a useful index for the classification. Moreover, the average sparse coefficient was used as classification index in LSRNN classifier and was demonstrated to be able to improve the performance of classification in the previous study [20]. Based on the previous study [20], this study also used the average of all coefficients related to a class as the classification criterion of SRC. For the SRC with an average coefficient (SRC-AVE) method, test sample *y* is assigned to an object class that has the maximal average value of the corresponding coefficients.

(6) $$\begin{array}{c} I\left(\;y\;\right)=\arg\underset{i}{\max}\;S_{i}\left(\;y\;\right)=\arg\underset{i}{\max}\;ave\left(\;\delta_{i}\left(\;\hat{x}\;\right)\;\right), \end{array}$$

where *S*_*i*_(*y*) is the mean of all coefficients from class *i*.

To solve the insufficiency of the training data, the unlabeled testing data can be used to update the training data. However, it is challenging for self-training learning to choose reliable unlabeled samples and guarantee the accuracy of the updated labels [26]. For SRC-AVE, the predication is usually more reliable if the average coefficient of the predicated class is much larger than that of the other classes. Based on this criterion, we investigated a method to measure the predication reliability of the testing sample. First, a distance *d*_*m*_ for the *m*th test sample *y*_*m*_ is defined in

(7) $$\begin{array}{c} {d_{m}=S}_{I}\left(\;y_{m}\;\right)-\frac{\sum_{j=1,j\neq I}^{K}S_{j}\left(y_{m}\right)}{K-1}, \end{array}$$

where *S*_*I*_(*y*_*m*_) is the mean of the coefficients from predicted class *I* and *S*_*j*_(*y*_*m*_) is the mean of the coefficients from the other classes. *K* is the total number of classes. The distance *d*_*m*_ measures how far the average coefficient *S*_*I*_(*y*_*m*_) of class *I* is from the mean of *S*_*j*_(*y*_*m*_) of the other *k* − 1 classes. If *d*_*m*_ is large enough, the predicated class label should be true with a high confidence. Thus, it is necessary to set a threshold to determine *d*_*m*_. Given the variability across the testing samples, the threshold is set as the mean of distances *d*_*i*_ (*i* = 1 ⋯ *m* − 1) of all the previous testing samples as follows:

(8) $$\begin{array}{c} {\text{threshold}}_{m}=\alpha \frac{\sum_{i=1}^{m-1}d_{i}}{m-1},\;\text{s.t.}\;\;m>1, \end{array}$$

where *m* corresponds to *m*th testing data. The threshold_*m*_ is fully determined by the previous *m* − 1 testing samples and reflects the average difference between the predicted class and the other unpredicted classes in the testing data. The coefficient *α* can be used to adjust the threshold level. In general, coefficient *α* can be set to 1 when the training data and testing data are from the same classes. When the training and testing data are not from the same classes, coefficient *α* can be set to less than 1 so that more testing data can be used to update the training data. For the first testing data, the training data cannot be updated by default. If the distance *d*_*m*_ of the *m*th testing sample is larger than threshold_*m*_, the predicated class label is considered reliable and the *m*th testing sample *y*_*m*_ is added to dictionary *A* as a new column. Dictionary *A* and training label *L* are replaced with *A* = [*A*, *y*_*m*_] and *L* = [*L*, *I*], respectively. Based on the updating criterion, we propose the semisupervised SRC-AVE (semiSRC-AVE) algorithm that combines self-training and SRC-AVE.  Algorithm 1 illustrates the semiSRC-AVE procedure.

## 4. Experiments and Results

In this section, we evaluated the effectiveness and robustness of semiSRC-AVE using simulated and real fMRI data. Moreover, the performance of semiSRC-AVE was compared to that of SRC-AVE and the other three self-training classifiers. The three classifiers were naïve semiSRC-AVE that updates the training data using naïve strategy, RSE-OLDC, and RSE-OSRC-AVE that used the SRC-AVE to replace the LDC of RSE-OLDC. The code of the GPSR algorithm was downloaded from http://www.lx.it.pt/~mtf/GPSR/, and the codes of SRC, SRC-AVE, semiSRC-AVE, and RSE-OSRC-AVE algorithms were written based on the core GPSR algorithm. In this paper, all the computations were performed on a computer with AMD Phenom(tm) II X4 B97 processor, CPU 3.20 GHz, and RAM 8 GB.

### 4.1. Simulated Experiments

#### 4.1.1. Generation of Simulated Data

Two groups of datasets were generated to investigate the performance of semiSRC-AVE in this section. It was assumed that each group of datasets included two runs and each run contained three tasks.

**Figure 1:**
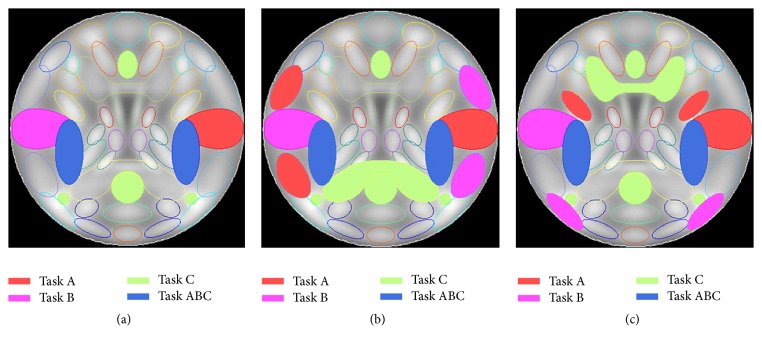
Regions of interest that are engaged in the three tasks for the first group of simulated datasets (a), the first run of the second group of datasets (b), and the second run of the second group of datasets (c).

The first group was generated by expressing the observations as the product of the time courses and super-Gaussian sources using the MATLAB toolbox SimTB [27]. For each run, we assumed that the simulated data from each subject contained 27 spatial sources. Each source that had 50 × 50 voxels with a baseline intensity of 800 was independently translated, rotated, and contracted or expanded to simulate the intersubject differences.  Figure 1(a) presents the regions of interest (ROI) that were activated by each task. The red/purple/green regions were assumed to be activated by tasks A/B/C, and the amplitude of each task block was set to 1.5 relative to the unique events whose amplitudes were 1. The blue regions were assumed to be engaged in the three tasks jointly with the amplitude of 2. There were twelve 40 s task blocks that alternated with twelve 20 s rest blocks with a TR of 2 s in each run, four blocks per task. The simulated fMRI response of each task that was derived from the convolution of the stimulus paradigm and the hemodynamic response function (HRF) was added to the corresponding ROIs. The simulated fMRI responses of the other task-unrelated sources were derived from the convolution of the unique events and HRF. Head motion was added with a translation of less than 2% of the entire image and a rotation less than 5 degrees. Each of the 12 subjects had nine levels of contrast-to-noise ratios (CNR) that varied from 0.08 to 0.16 with an increment of 0.01. Thus, the first group of simulated datasets contained 108 (12 × 9) datasets. The order of the task blocks was randomized across the nine noise levels. At each noise level, the simulated datasets of 12 subjects had the same order of task blocks.

The second group of datasets was used to investigate the performance of semiSRC-AVE for cross-decoding that tested generalization to novel stimuli or tasks. In cross-decoding, a decoder is trained with one set of stimuli and then tested with another, or the task eliciting the response patterns is changed [28]. For the second group, it was assumed that the tasks in the training runs were different from the tasks in the testing runs and the regions activated by the tasks in the training runs were not the same as those in the testing runs. The second group of simulated datasets was generated in the same way as the first group, except that the activated ROIs were different. Figures 1(b) and 1(c) present the ROIs that were activated by the three tasks in the first run and second run, respectively.

#### 4.1.2. Feature Selection

Each subject's first run was used as the training samples, and the second run was used as the testing samples for all the simulated datasets. The correlation between the time course of each voxel and the reference function was calculated. The reference function was derived from the convolution of the paradigm of the three tasks and HRF. The top 1000 voxels with the highest correlations were selected as features. The testing data in the second run used the same features as the training data. As a pragmatic approach, we fixed the number of voxels rather than the level of the correlations that may fluctuate across different datasets. The time course of each voxel and the spatial pattern of each time point were then normalized to a zero mean and unit variance.

#### 4.1.3. Comparison of SRC and SRC-AVE

The first group of datasets was used to compare the performances of SRC and SRC-AVE. Three-class classifiers of SRC and SRC-AVE were trained from the training data (the first run) and applied to the testing data (the second run). For each subject in each group, the accuracy was calculated at each noise level using the ratio between the number of testing samples that were correctly classified and the total number of testing samples. The mean accuracy of each classifier across the 12 subjects was obtained at each noise level for each group of datasets.

#### 4.1.4. Comparison of the Semisupervised Learning Methods

Both the first and second groups of datasets were used to investigate the performances of semiSRC-AVE in the case of different noise levels and cross-decoding. Because the advantage of SRC-AVE over SRC in fMRI-based decoding was demonstrated in the above section, only SRC-AVE was used to be compared with the semisupervised learning method in the following simulated and real fMRI experiments. Two-class (tasks A and B) and three-class SRC-AVE, semiSRC-AVE, naïve semiSRC-AVE, RSE-OLDC, and RSE-OSRC-AVE classifiers were trained from the training data (the first run) and applied to the testing data (the second run). The mean accuracy of each classifier across the 12 subjects was obtained at each noise level for each group of datasets. To examine the difference of the classification accuracies between the proposed semiSRC-AVE and the other four methods, the nonparametric Wilcoxon signed rank tests for paired samples were performed. Moreover, the computation time of each three-class classification for the first group of dataset was recorded to compare the time efficiency of all the five methods. The mean time across 12 subjects of all noise levels was calculated.

#### 4.1.5. Determination of Parameter *α* in SemiSRC-AVE

Parameter *α* was set to 1 in the first group of datasets because the training and testing data were from the same tasks. For the second group of datasets that used different tasks in the training and testing data, the optimal value of parameter *α* was determined using the simulated datasets. The three-class semiSRC-AVE classifiers with different *α* values were trained using each subject's training data in the second group. Parameter *α* was varied from 0.5 to 1 with an increment of 0.1. For each *α*, the mean accuracy across all subjects was obtained for each noise level, and then the mean accuracy across all noise levels was obtained. The *α* value with the highest accuracy was selected as the optimal value for the datasets with different training and testing tasks.

### 4.2. Results of Simulated Experiments

#### 4.2.1. Comparison of SRC and SRC-AVE

**Figure 2:**
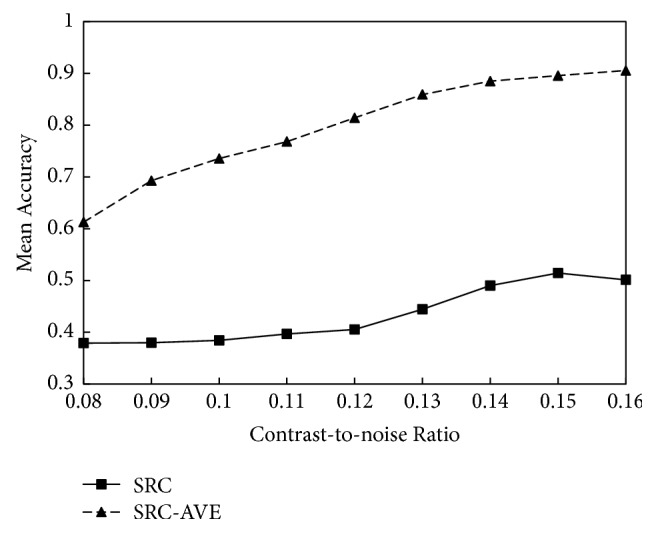
The mean classification accuracies of SRC and SRC-AVE at various CNR levels.

Figure 2 shows the mean accuracies of SRC-AVE method compared with those of the conventional SRC at all the CNR levels. The mean accuracies of the two methods increased as the CNR levels increased (see Figure 2). The classification accuracies of SRC are much lower than those of SRC-AVE at all the noise levels.

#### 4.2.2. Comparison of the Semisupervised Learning Methods

**Figure 3:**
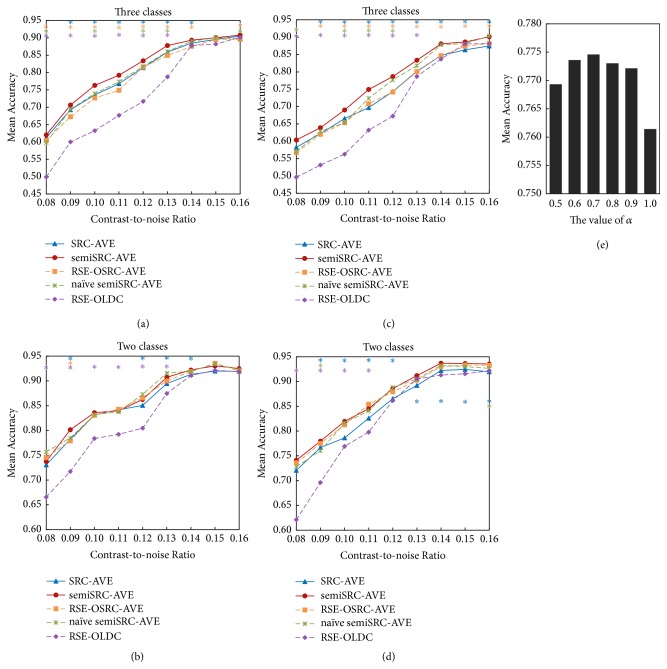
Mean accuracies of SRC-AVE, semiSRC-AVE, naïve semiSRC-AVE, RSE-OLDC, and RSE-OSRC-AVE classifiers. (a) Three-class performances of the simulated datasets with the same training and testing tasks. (b) Two-class performances of the simulated datasets with the same training and testing tasks. (c) Three-class performances of the simulated datasets with different training and testing tasks. (d) Two-class performances of the simulated datasets with different training and testing tasks. (e) Determination of parameter *α* in semiSRC-AVE. The asterisk *∗* represents *p* < 0.05. The color of asterisk indicates the comparison between semiSRC-AVE and the method having the same color as the asterisk in each panel.

Figures 3(a)-3(b) show the mean accuracy of SRC-AVE and the four semisupervised methods at different CNR levels for the first group of simulated datasets. For the three-class classification, RSE-OLDC showed the worst performance, and semiSRC-AVE showed the best performance at all noise levels (see Figure 3(a)). SemiSRC-AVE showed significantly higher accuracy than SRC-AVE, RSE-OSRC-AVE, and RSE-OLDC at most noise levels and significantly higher accuracy than naïve semiSRC-AVE at the middle noise levels. For the two-class classification, the accuracy of RSE-OLDC was the lowest, while the performances of semiSRC-AVE, RSE-OSRC-AVE, and naïve semiSRC-AVE showed slight differences. The accuracies of SRC-AVE were lower than those of semiSRC-AVE, RSE-OSRC-AVE, and naïve semiSRC-AVE at most noise levels (see Figure 3(b)).

Figures 3(c)-3(d) show the performances of the five classifiers for cross-decoding the second group of simulated datasets with different training and testing tasks. For the three-class classification, semiSRC-AVE produced the highest accuracy while RSE-OLDC produced the lowest accuracy among the five methods (see Figure 3(c)). SemiSRC-AVE showed significantly higher accuracy than SRC-AVE, RSE-OSRC-AVE, and RSE-OLDC at most noise levels and significantly higher accuracy than naïve semiSRC-AVE at the middle noise levels. For the two-class classification, the performance of RSE-OLDC remained the worst, while the performances of semiSRC-AVE, RSE-OSRC-AVE, and naïve semiSRC-AVE only showed slight differences (see Figure 3(d)). The semiSRC-AVE significantly outperformed supervised-learning SRC-AVE at most noise levels. In contrast to the datasets with the same training and testing tasks, the accuracy of SRC-AVE was reduced to a larger extent than that of semiSRC-AVE for cross-decoding the datasets with different training and testing tasks (see Figure 3).

#### 4.2.3. Determination of Parameter *α* in SemiSRC-AVE

For cross-decoding the dataset with different training and testing tasks, the mean accuracies of semiSRC-AVE using six different *α* values are presented in Figure 3(e). It can be seen that the mean accuracy was the highest when parameter *α* was set to 0.7. Therefore, 0.7 was used as the optimal value of parameter *α* in cross-decoding the simulated and real fMRI datasets with different training and testing tasks.

#### 4.2.4. Time Efficiency Analysis

Table 1 lists the computation time of the five three-class classifiers for the first group of simulated datasets. The computation time of semiSRC-AVE was close to that of supervised-learning classifier SRC-AVE. RSE-OSRC-AVE, and RSE-OLDC took much more computation time than SRC-AVE, semiSRC-AVE, and naïve semiSRC-AVE.

### 4.3. Real fMRI Experiment

The real fMRI data used in this study were the same as those used in our previous study [29]. For readability, the main points are repeated here.

#### 4.3.1. Datasets

Fourteen right-handed college students (age: 22.2 ± 1.9 years, eight females) participated in this study. The fMRI data were acquired using a 3-T Siemens scanner equipped for echo planar imaging (EPI) at the Brain Imaging Center of Beijing Normal University (TR = 2000 ms; TE = 30 ms; 32 slices; voxel size = 3.125 × 3.125 × 3.84 mm; flip angle (FA) = 90; FOV = 190 × 200 cm). In addition, a high-resolution, three-dimensional T1-weighted structural image was acquired (TR = 2530 ms; TE = 3.39 ms; 128 slices; FA = 7; resolution = 1 × 1 × 1.33 mm).

The experiment was conducted in a block design and consisted of eight runs. Each run included four 24 s task blocks that were alternated with five 12 s resting blocks. Visual stimuli belonging to four categories (i.e., house, face, car, and cat) corresponded to the four tasks in each run and were separately displayed for 500 ms, followed by a 1500 ms blank screen.

The data preprocessing was performed using Statistical Parametric Mapping (SPM8) (http://www.fil.ion.ucl.ac.uk/spm/software/spm8/). For each subject, the first three volumes of each run were removed due to the instability of the initial scanning of each run. The functional images of each subject were realigned to correct for head motion, spatially normalized into the standard Montreal Neurological Institute (MNI) template space, resliced into 3*∗*3*∗*4 mm^3^ voxels, and spatially smoothed using an 8 mm full-width-at-half-maximum (FWHM) Gaussian kernel.

#### 4.3.2. Comparison of Classifiers

*(a) Datasets with the Same Training and Testing Tasks. *A generalized linear model (GLM) was applied to each subject's training data to estimate the brain regions that were activated by each task using SPM8. The significance level was set as *p* < 0.001 and was uncorrected. A brain mask that included the fusiform gyrus, inferior temporal gyrus, inferior occipital gyrus, and middle occipital gyrus was generated using the WFU Pickatlas toolbox (http://www.fmri.wfubmc.edu). The union of the voxels that were activated by each task within the mask was selected as features. The testing data used the same features as the training data. For each dataset, the linear drift was removed using the spm_detrend function in SPM8. The time series of each feature and the spatial pattern of each scan were normalized to a zero mean and unit variance.

For each subject, a twofold cross validation was performed. In the first fold, the first four runs were used as the training runs, and the last four runs were used as the testing runs, and vice versa in the second fold. Four-class, three-class, and two-class classifiers of SRC-AVE, semiSRC-AVE, naïve semiSRC-AVE, RSE-OLDC, and RSE-OSRC-AVE were trained from each subject's training data separately. Each classifier was applied to each test volume to determine the task state. The mean accuracy across the 14 participants in the twofold was obtained for each classifier. To examine the difference in the classification accuracies between any two methods, the nonparametric Wilcoxon signed rank tests for paired samples were performed. For each method, the computation time of the four-class classification was recorded and the mean time across the 14 subjects was calculated.

 (*b) Cross-Decoding Datasets with Different Training and Testing Tasks*. To further explore the performance of semiSRC-AVE for cross-decoding, we regenerated the training and testing datasets. It should be noted that cat and human face are animate objects with sense organs while house and car are inanimate objects. Therefore, the volumes of the house and cat tasks in eight runs consisted of the training datasets, and the volumes of the car and face tasks in eight runs consisted of the testing datasets for each subject. The GLM was applied to the training data, and any voxels that were activated by at least one of the two tasks (i.e., house and cat) within the mask were selected as features. After SRC-AVE, semiSRC-AVE, naïve semiSRC-AVE, RSE-OLDC, and RSE-OSRC-AVE classifiers were trained from the training samples, they were applied to the testing volumes to predict their task states (car versus face). The mean accuracy of each classifier across the 14 subjects was obtained. Moreover, we also performed classification by using the volumes of the car and face tasks as the training data and the volumes of the house and cat tasks as the testing data. The feature selection and classifications were performed in the same way as described above. The nonparametric Wilcoxon signed rank tests for paired samples were performed to examine the differences between any two methods.

### 4.4. Results of Real fMRI Experiment

#### 4.4.1. Comparison of Classifiers

**Figure 4:**
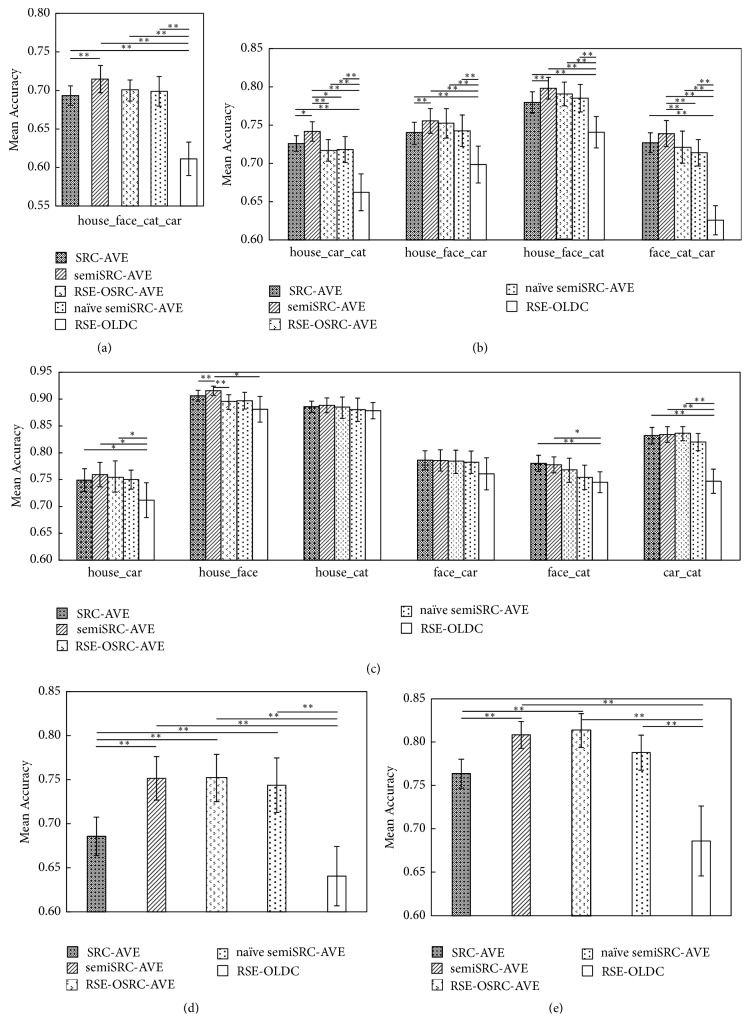
Mean accuracies of real fMRI data with same (a–c) and different (d-e) training and testing tasks using the SRC-AVE, semiSRC-AVE, naïve semiSRC-AVE, RSE-OLDC, and RSE-OSRC-AVE classifiers. (a) Two-class classifications. (b) Three-class classifications. (c) Four-class classifications. (d) Training data consisting of house and cat tasks. (e) Training data consisting of face and car tasks. The symbols “*∗∗*” represent *p* < 0.05 and the symbol “*∗*” represents *p* < 0.1.

*(a) Datasets with the Same Training and Testing Tasks*. Figures 4(a)–4(c) display the mean accuracies of the one four-class, four three-class, and six two-class classifications of SRC-AVE and the four semisupervised learning methods. SemiSRC-AVE showed the highest accuracy in most cases, and RSE-OLDC showed the lowest accuracy in all cases among the five methods. In contrast to SRC-AVE, semiSRC-AVE produced a significantly higher accuracy for the four-class classification, three three-class classifications, and one two-class classification. No significant differences were found between the performances of naïve semiSRC-AVE/RSE-OSRC-AVE and SRC-AVE for all classifications. Moreover, the accuracy of semiSRC-AVE was significantly higher than that of RSE-OSRC-AVE for house versus car versus cat, face versus cat versus car, and house versus face and significantly higher than that of naïve semiSRC-AVE for house versus car versus cat and face versus cat versus car.

*(b) Cross-Decoding Datasets with Different Training and Testing Tasks*. Figures 4(d)-4(e) show the results of the two-class classifications of SRC-AVE and the four semisupervised learning methods for cross-decoding datasets with different training and testing tasks. When the volumes of the house and cat tasks were used as the training data, the three semisupervised learning methods, including semiSRC-AVE, naïve semiSRC-AVE, and RSE-OSRC-AVE, produced significantly higher accuracies compared to that of SRC-AVE (see Figure 4(d)). The accuracy of RSE-OLDC was significantly lower than those of the other four methods. When the volumes of the car and face tasks were used as the training data, semiSRC-AVE and RSE-OSRC-AVE showed a significantly higher performance than that of SRC-AVE, and RSE-OLDC showed a significantly worse performance than those of the other four methods (see Figure 4(e)).

#### 4.4.2. Time Efficiency Analysis

Table 2 presents the mean computation time of the five four-class classifiers for the real fMRI datasets. The computation time of semiSRC-AVE was close to that of SRC-AVE and was less than that of RSE-OSRC-AVE and RSE-OLDC.

## 5. Discussion

The present study proposed the semisupervised learning semiSRC-AVE method to improve the decoding performance of SRC. Our major findings are as follows: (1) the semiSRC-AVE method can significantly improve the performance of SRC-AVE, particularly for cross-decoding; and (2) in contrast to RSE-OSRC-AVE, naïve semiSRC-AVE, and RSE-OLDC, semiSRC-AVE method shows better performance for multiclass classification and comparable performance for two-class classification.

SRC exhibited state-of-the-art classification performances in previous studies of image classification and recognition [3]. However, in this study, SRC produced a very low classification accuracy when it was applied to the fMRI data. There are two possibilities that may result in the low performance of SRC for fMRI-based decoding. One possibility is the complicated and high noises in the fMRI data. Generally, fMRI data contain various noises, such as thermal noise, system noise, motion and physiological noise, non-task-related neural variability, and behavioral and cognitive variability [29]. Signals in fMRI data are much weaker than noises. Moreover, behavioral and cognitive variability may lead to changes in arousal and attention over time. Evoked brain activity may change in each trial, even in the same types of trials. Another possibility may be attributed to the hemodynamic response. After the stimulus onset, the hemodynamic response exhibits an initial dip, rises to a peak value after approximately 6 seconds, and then falls to the baseline level [29]. There is a delay between the peak and the stimulus onset, indicating that the initial volumes of each task block may show very different brain activity patterns than the volumes in the middle of the blocks, although the volumes in the same task blocks respond to the same type of stimulus. Both the noises and hemodynamic responses can result in large variabilities in the brain activity that is evoked by each trial. For the sparse representation model, testing sample *y* is assumed to be linearly represented by the training samples of the same class. Because of the high variability in the training and testing samples of the same class in fMRI data, the testing samples cannot be well represented by the training samples and coefficient vector *x* may not be very sparse. Accordingly, SRC does not perform well in decoding brain states from fMRI data.

The results of the simulated data showed that SRC-AVE had a much better performance than SRC. It should be noted that the test samples are generally represented by the training samples from the same class with larger positive coefficients and smaller negative coefficients than the training samples from the other classes. Thus, the average of all positive and negative coefficients can be used as a criterion to determine the testing samples, which is consistent with the previous study that demonstrated that class-specific average sparse coefficients were useful to improve the performance of classification [20]. Moreover, our results further indicated that the average coefficient worked better than the reconstruction error for SRC in fMRI-based decoding.

Both the simulated and real fMRI datasets indicated that the proposed semiSRC-AVE effectively improved the performance of SRC-AVE, particularly for the multiclass classifications. SRC-AVE method used fixed training dataset while semiSRC-AVE gradually updated training dataset using the new testing samples with high confidence. The performance of SRC-AVE was easily affected by the insufficient labeled training samples because limited training dataset tended to increase the generalization error of classifiers. In contrast, the performance of semiSRC-AVE was improved due to gradually enlarged training dataset. Meanwhile, the distance between the predicted class and the other classes was calculated to measure the confidence of the predicated label of each test sample for semiSRC-AVE. The threshold of the confidence was determined by the previous testing samples rather than a fixed value. Because the threshold was adaptive to the changes of the testing samples, it was helpful to the selection of the testing samples with high confidence. The results indicated that the selected testing samples using the proposed strategy were reliable and contributed to the improvement of semiSRC-AVE.

For cross-decoding, compared to SRC-AVE, the advantages of semiSRC-AVE were more prominent. Because the training and testing samples were not entirely from the same distribution, the testing samples cannot be well represented by the training data, which may greatly affect the performance of SRC-AVE. For semiSRC-AVE, the training dataset was updated by the testing data. The dictionary that was adaptive to the testing data represented the testing samples better than the fixed dictionary. Accordingly, semiSRC-AVE worked much better than SRC-AVE when the training and testing tasks were different. In contrast to SRC-AVE, semiSRC-AVE showed better generalization to novel stimuli or tasks.

Among the four semisupervised learning methods, RSE-OLDC showed the worst performance and semiSRC-AVE showed the best performance in both the simulated and real fMRI experiment. The worst performance of RSE-OLDC may be attributed to the worse performance of LDC than SRC-AVE. In contrast to the naïve semiSRC-AVE and RSE-OSRC-AVE, the semiSRC-AVE showed better performance for multiclass classification and comparable performance for the two-class classification. In contrast to multiclass classification, two-class classification generally has higher prediction accuracy and produces more reliable predicated labels of the testing samples. Therefore, the confidence determination for the testing samples may have a minor effect on the two-class classification of the semisupervised learning methods, which possibly resulted in the comparable performance of the naïve semiSRC-AVE and semiSRC-AVE in the two-class classification. In contrast, the confidence determination of semiSRC-AVE played an important role in selecting reliable testing samples to update the dictionary in the multiclass classification. Thus, semiSRC-AVE outperformed naïve SRC in the three-class classification. Moreover, the results also indicated that the confidence determination strategy of semiSRC-AVE was simpler, more time-saving, and more efficient compared to the random subspace ensemble strategy of RSE-OSRC-AVE.

It should be noted that there is a balance between the update accuracy and the number of updated samples. If the threshold of the confidence determination is increased to improve the update accuracy, the number of updated samples will be reduced and vice versa. The small number of updated samples may only have a slight impact on the classifiers. It is critical to maintain a balance between the accuracy and the proportion of updates for semisupervised learning. We set parameter *α* to adjust the threshold for different types of datasets. Our simulated and real fMRI datasets indicated that a threshold with *α* = 1 worked well for datasets with the same training and testing classes. For certain specific datasets, properly adjusting parameter *α* may achieve better results. For cross-decoding, the amount of updated samples becomes more important due to the lack of test-like samples in training dataset. Therefore, parameter *α* was set to 0.7, which was demonstrated to be the optimal value by cross-decoding the simulated data with different training and testing tasks in the study. The lowered parameter *α* allowed more testing data to update the training data. Both our simulated and real fMRI experiments indicated that the optimal value 0.7 was good for data with different training and testing classes.

## 6. Conclusion

In this study, we demonstrated the robustness and feasibility of the semisupervised learning semiSRC-AVE using both simulated and real fMRI data. The results indicated that semiSRC-AVE showed significantly better performance than SRC-AVE. In addition, semiSRC-AVE performed better than naïve semiSRC-AVE, RSE-OSRC-AVE, and RSE-OLDC for multiclass classifications. For the two-class classification, semiSRC-AVE did not show prominent advantages over the other three semisupervised learning methods. Therefore, it is essential to further investigate the optimal update strategy that is suitable to the two-class classification in the future studies. Moreover, the proposed update strategy in this study can be easily extended to the other supervised-learning classifiers.

## Figures and Tables

**Algorithm 1:**
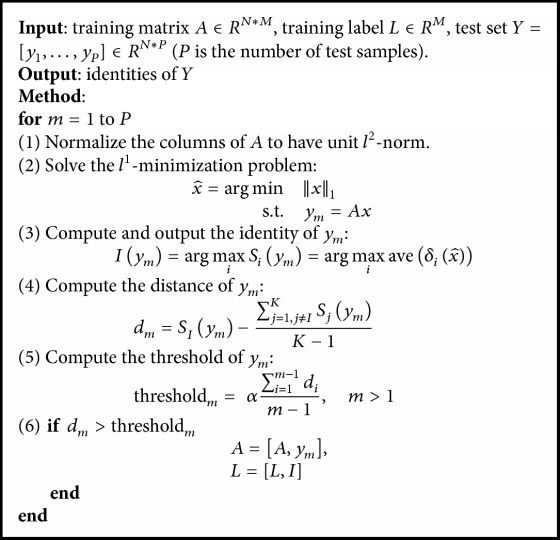
semiSRC-AVE.

**Table 1:** Mean computation time of the five three-class classifiers for the simulated datasets.

	SRC-AVE	SemiSRC-AVE	RSE-OSRC-AVE	Naïve semiSRC-AVE	RSE-OLDC
Time (second)	3.55	5.83	46.70	5.88	30.87

**Table 2:** Computation time of five classifiers for real fMRI datasets.

	SRC-AVE	SemiSRC-AVE	RSE-OSRC-AVE	Naïve semiSRC-AVE	RSE-OLDC
Time (second)	2.30	2.71	17.57	3.26	7.90

## Data Availability

The datasets used and analysed during the current study are available from the corresponding author on reasonable request.
